# Atypical hyperglycemia presentation suggests considering a diagnostic of other types of diabetes: first reported GCK-MODY in Perú

**DOI:** 10.1186/s40842-019-0091-x

**Published:** 2020-01-14

**Authors:** Juan Carlos Lizarzaburu-Robles, Juan Carlos Gomez-de-la-Torre, María del Carmen Castro-Mujica, Flor Vento, Sofia Villanes, Elizabeth Salsavilca, Chris Guerin

**Affiliations:** 1 Hospital Central de la Fuerza Aérea del Perú, Lima, Peru; 2 Asociación para la Prevención, Educación e Investigación en Diabetes – APREDIAB, Lima, Peru; 3 Sequence Reference Lab, Lima, Peru; 4 Advanced Metabolic Care and Research San Diego, San Diego, USA

**Keywords:** Hyperglycemia, Diabetes, GCK-MODY, Latin-America

## Abstract

**Background:**

Prevalence of maturity-onset diabetes of the young (MODY) is estimated between 1 and 2% of all diabetes cases. In Latin-America little information has been described about the frequency of the disease, perhaps due to limited access to genetic studies.

**Case presentation:**

We present the case of a male patient with a history of two years of fatigue, mild hyperglycemia and intermittent polyuria, accompanied by a recent history of weight loss. He was diagnosed initially as type 2 diabetes, but in the follow-up as a patient with type 1 diabetes. He required relatively low doses of insulin and was evaluated in the endocrinology service at a hospital in Lima. The results of glucose, insulin and C-peptide in the oral glucose tolerance test (OGTT) performed were not consistent with a type 1 diabetes. Moreover, the age of the patient and the clinical characteristics did not strongly suggest a diagnosis of type 2 diabetes either. These clinical features had prompted us to carry out the genetic study. The genetic test performed with a genetic MODY panel through a massive sequencing. Heterozygous pathogenic for a variant in *GCK* gene was found c.629C>T p.(Thr210Met). His parents were negative for this variant after performed the genetic test.

**Conclusions:**

This is the first case of MODY for a pathogenic variant in the *GCK* gene reported in Perú. The genetic evaluation of a clinical suspicion of MODY is important to confirm the diagnosis and establish an adequate treatment in patients.

## Background

Maturity-Onset Diabetes of the Young (MODY) is a rare monogenic disease which accounts for 1–2% of all diabetes cases [1, 2]. Recently 14 genes associated with MODY have been proposed and the clinical characteristics differ according to the genetic etiology [3]. MODY should be suspected in non-obese subjects, onset before 25 years with mild hyperglycemia and absence of Diabetic ketoacidosis (DKA) [1, 2, 4]. GCK-MODY or MODY2 is one of the most frequent subtypes described and is caused by mutations in the *GCK* gene inherited in an autosomal dominant manner. It is due to mutations in the *GCK* gene encoding glucokinase, which catalyzes the conversion of glucose to glucose-6-phosphate, an important step in glucose metabolism and the regulation of insulin secretion [3, 5].

To date, over 600 different GCK-MODY mutations have been reported and it has been described in the literature in a variety of ethnic groups, most of them in Europeans countries (Italy, France, United Kingdom and Spain, according to the literature) and in North America [1, 3, 5–7]. There is little information about the frequency of MODY in Latin America, and it is lower when we try to find information in countries with Native-American or American Indian population. Nevertheless, MODY may be more frequent than previously assumed. Also, the proportion of cases caused by a “de novo” pathogenic variant is unknown for most MODY-related genes. There are a limited number of case reports which describe de novo variants in *GCK* gene [8]. We report the first case of MODY with a pathogenic variant *GCK*, with a complete genetic analysis, in Perú.

## Case presentation

**Fig. 1:**
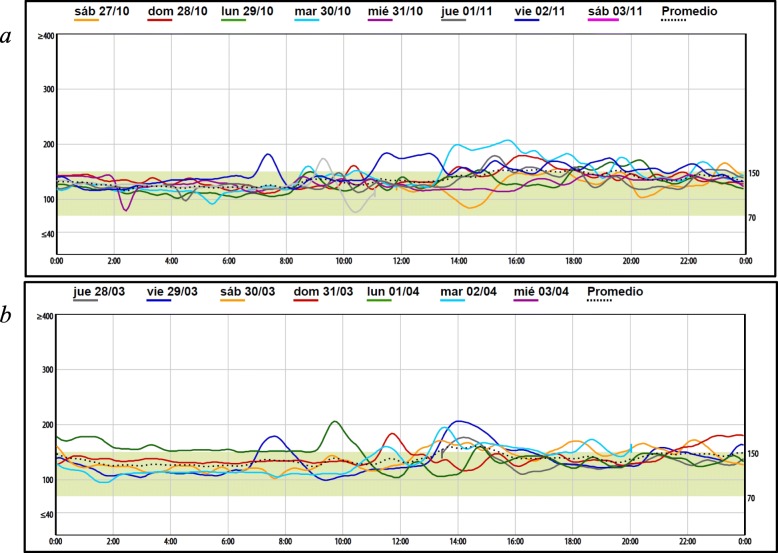
Patient glucose-monitoring (iPro2) with Insulin (**a**) and oral agents (**b**) *Dash line (*^***…***^*) Represent one week references glycemic control*

A 15 years old male diagnosed with type 2 diabetes was referred to an endocrinology outpatient clinic. He was diagnostic at age 13 with an A1c control in 6.8% and initiated treatment with metformin twice a day. After two years of irregular follow up, the patient presented with history of 2 kg weight loss, fatigue, mild polydipsia and polyuria but without signs of insulin resistance, such as acanthosis nigricans and/ or obesity. His weight was 52 kg (BMI between the 10th and 25th percentiles for age and gender), Hb: 14.6 g/dL, fasting glucose: 132 mg/dl, Hb A1c: 6.4%, creatinine clearance: 181.61 ml/min, C-peptide: 1.63 nmol/L, total cholesterol:166, LDL: 114.2 mg/dl, Triglycerides: 64 mg/dl and HDL: 39 mg/dl. Also, he had TSH: 1.43 mIU/L and FT4: 1.21mμg/dl. He had Glutamic acid decarboxylase (GAD) antibodies and anti-islet cell antibodies which were negative. In his medical history, his birth weight was 3.06 kg with no family first degree relative with history of Diabetes. We decided to initiate insulin treatment (12 units/day of Levemir and 01unit of pre-prandial lispro) with good clinical response. The patient did not demonstrate hypoglycemia during this period. Both situations suggested the diagnostic of type 1 diabetes and the probability that he was on a spontaneous period of partial clinical remission (honeymoon phase) [9, 10]. In the follow-up period over the next 6 months, the insulin requirement did not change, and he maintained good glucose control levels (Fig. 1a). Hb A1c control was 6.3%.

**Fig. 2:**
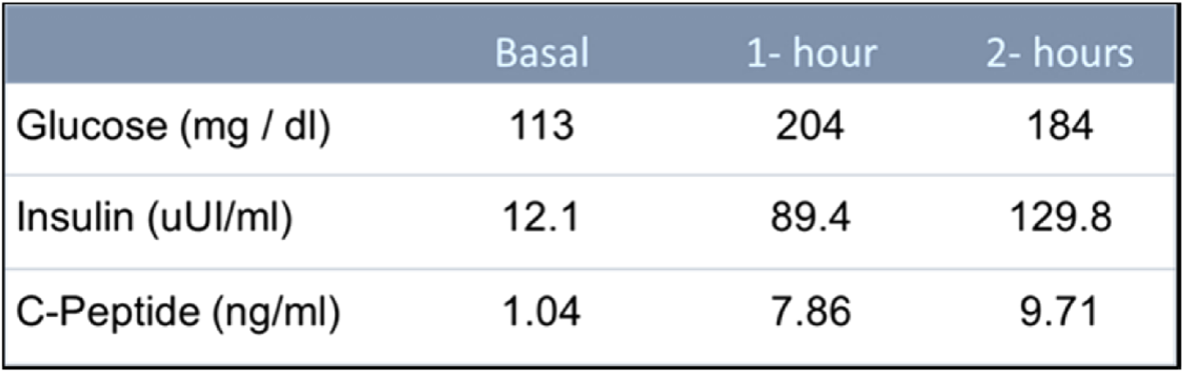
OGTT include insulin and “C” peptide results

The low insulin-dose requirement in a long time and his non-acute presentation, in the childhood, of diabetes was unusual for type 1 diabetes. We decide to request an Oral Glucose Tolerance Test (OGTT) with measure of Glucose (mg / dl), insulin (uUI/ml) and C-peptide (ng/ml) to evaluate pancreatic response. The following results for the test we see in the Fig. 2.

**Fig. 3:**
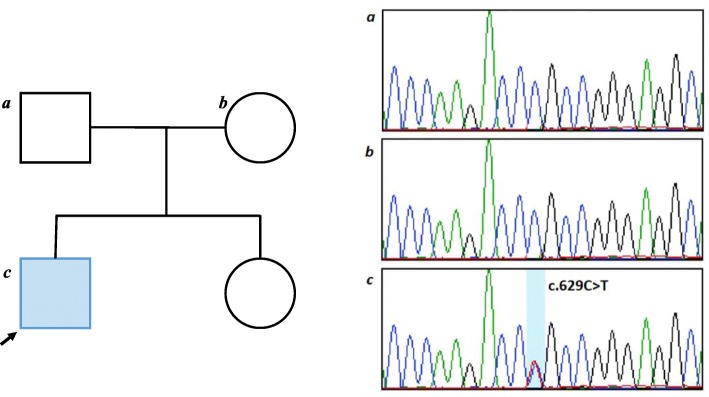
Heterozygous pathogenic variant c.629C>T p.(Thr210Met). in *GCK* gene

These results were not typical of either type 1 or type 2 diabetes and supported further evaluation. In addition, we performed an OGTT on his parents, with normal results. We considered appropriate to perform a test for diagnosis of MODY. MODY panel was performed in the next follow up, by next generation sequencing identifying a pathogenic variant c.629C>T p.(Thr210Met). in heterozygous in *GCK* gene confirmed by Sanger sequencing according to the American College of Medical Genetics (Fig. 3). However, this variant has been previously described as associated with MODY diabetes [11, 12]. The biochemical studies show this variant causes the reduction of GCK enzyme activity. This result confirmed the diagnosis of GCK-MODY. The specific molecular analysis for GCK also was performed in his parents. The result was negative for this variant, which suggested that it was a “de novo” variant.

With the result of genetic analysis, we suspended insulin. We decided to initiate metformin (850 mg) twice/day. The clinical response was excellent as well as the initial treatment (Fig. 1b). We also suggested life-style change with nutritional follow-up.

In the follow-up period to asses control, continuous glucose monitoring was performed to evaluate glucose levels. We see the initial response to insulin treatment during this period in Fig. 1a. A second report of continuous glucose monitoring was performed during oral medication treatment (Fig. 1b). Currently, the patient continues with only oral medication (metformin), and he increased his weigh with optimal clinical control. His most recent Hb A1c was 6.2%.

## Discussion

MODY is considered an infrequent disease, and it has been described in several populations and ethnic groups worldwide, more commonly in Europe and North America [1, 5, 7]. A recent publication about GCK-MODY in the US National Monogenic Diabetes Registry, shows that the majority of Registry participants with a GCK-MODY phenotype self-identified as Caucasian and there was underrepresentation of a number of ethnic minorities when compared to the greater US population and ethnicity-specific diabetes rates [5].

This is the first reported case in Perú, and in a Mestizo-Peruvian ethnic group, which consist in a population with Native-American and European ancestries [13]. Other countries in Latin America, such as Argentina and Chile, also have reported cases mainly associated with GCK-MODY, which seems to be frequent in our region [6, 7, 14]. It should be noted that not all countries in Latin-American have the same ethnic groups.

Clinical suspicion should be our first step in the diagnosis of this type of diabetes [1, 2, 4]. In our case, initially we assume a Type 1 DM in a period of partial clinical remission or honeymoon phase. As it is known, insulin requirement decreases during this period [9, 10]. Identifying maturity-onset diabetes of the young (MODY) in pediatric populations is difficult. Misdiagnosis and unnecessary insulin treatment are commonly used [15]. However, the low doses insulin requirement in a long period of time, more than 06 months, and the absence of DKA diminished the possibility of type 1 diabetes [16]. On the other hand, the age at diagnosis, the absence of relatives with type 2 diabetes and obesity reduce the probability of type 2 diabetes. Treatment with metformin was considered as a benefit to improve insulin sensitivity [17, 18], because according to the TTOG performed (Fig. 2), the patient presented an insulin test profile that suggested insulin resistance.

Molecular genetic testing to determine the gene associated with MODY can include gene panels or unique gene analysis, as well as exome sequencing [3]. In our patient, the absence of previous reported cases in Perú and the little information about frequency of MODY types in the Latino population, led us to the determination of carrying out a MODY Panel genetic analysis. MODY *GCK* gene-variant suspicion was confirmed. *GCK* mutations cause a resetting of the glucose and is associated with mild hyperglycemia and low ranges of HbA1c (less than 7.6%), while the secretion and regulation of insulin is not completely altered [3, 4, 19]. The hyperglycemia in GCK-MODY is present throughout life but is usually asymptomatic and only detected when blood glucose is measured incidentally [20]. Complications are rare and medication has been shown to have minimal effect [1, 4, 21].

It was interesting to mention that parents were negative for the mutation in the genetic analysis. Thus, suggested that the mutation diagnosed might be “de novo”. Frequently, MODY diabetes is present in at least two consecutive generations, although this is not a good discriminator from young patients with Type 2 diabetes [20, 21]. Few de novo mutations have been reported, suggesting a low prevalence [6, 8]. Nevertheless, in the recent years the views on prevalence have been changing, and new publications suggest that de novo mutations could be more frequent than deduced from previously available [8]. A recent report in a Latin-American country, showed that 14% of GCK-MODY cases were de novo [6].

## Conclusion

This is the first case of Diabetes MODY with a pathogenic variant in the *GCK* gene reported in Perú. Heterogeneity of diabetes presentations should make us suspect MODY in patients who cannot be characterized as classical patients with type 1 or 2 Diabetes. Because of rarely tested of these cases, we have not a complete information about the frequency of Other specific types of diabetes in Latin America.

A complete medical evaluation should be the first step to classify the patient, however the genetic evaluation of a clinical suspicion of MODY diabetes is important to confirm the diagnosis and establish an adequate treatment in patients.

## Data Availability

The data used in this case report are available in the patient’s medical record and can be disclosed by the corresponding author on reasonable request.

## Abbreviations

GAD: Glutamic acid decarboxylase
GCK: Glucokinase
MODY: Maturity-Onset Diabetes of the Young
OGTT: Oral glucose tolerance test
