# Correction to: Atypical hyperglycemia presentation suggests considering a diagnostic of other types of diabetes: first reported GCK-MODY in Perú

**DOI:** 10.1186/s40842-020-0093-8

**Published:** 2020-02-17

**Authors:** Juan Carlos Lizarzaburu-Robles, Juan Carlos Gomez-de-la-Torre, María del Carmen Castro-Mujica, Flor Vento, Sofia Villanes, Elizabeth Salsavilca, Chris Guerin

**Affiliations:** 1 Hospital Central de la Fuerza Aérea del Perú, Lima, Peru; 2 Asociación para la Prevención, Educación e Investigación en Diabetes – APREDIAB, Lima, Peru; 3 Sequence Reference Lab, Lima, Peru; 4 Advanced Metabolic Care and Research San Diego, San Diego, USA

**Correction to: Clin Diab Endocrinol**

**https://doi.org/10.1186/s40842-019-0091-x**

It was highlighted that the original article [1] contained an error regarding the nomenclature in the gene described. This was incorrectly captured as c.629 T > C, p.Met210Thr. The correct form is c.629C > T p.(Thr210Met). This error appeared in the Case Presentation section of the Abstract, in the Case Presentation section in the main body of the article and in the legend of Fig. 3. The original article has been updated.
